# Eating Disorders in Children and Adolescent Males: A Peculiar Psychopathological Profile

**DOI:** 10.3390/ijerph191811449

**Published:** 2022-09-11

**Authors:** Anna Riva, Maria Pigni, Nunzia Delia Albanese, Mariella Falbo, Simona Di Guardo, Eleonora Brasola, Francesco Biso, Renata Nacinovich

**Affiliations:** 1 Child and Adolescent Mental Health Department, ASST Monza University of Milano-Bicocca, 20900 Monza, Italy; 2 Department of Business Engineering, Tor Vergata University of Rome, 00133 Rome, Italy

**Keywords:** eating disorders, males, children, adolescents, psychopathology

## Abstract

(1) Background: Eating Disorders (EDs) are severe psychiatric disorders with high rates of mortality, multiple medical and psychiatric comorbidities associated, and often chronic illness. Historically, EDs are among the most gendered of psychiatric illnesses, and male presentations have been perceived as rare and unusual. This perception resulted in the systematic underrepresentation of males in research on Eds, and as consequence, in a scarcity of research investigating clinical and psychological features in this population. (2) Methods: The present study aims to evaluate clinical and psychopathological features in a sample of 287 children and adolescents, 27 males and 260 females with EDs, in order to identify similarities and differences. (3) Results: Males were younger than females, with similar medical and clinical conditions, but a different distribution of typology of EDs in middle childhood and middle adolescents. The Eating Disorders Inventory-3, TAS-20 for alexithymia and CDI for depressive symptoms’ profiles are similar, while males showed higher scores at the global indexes of Symptom Checklist 90-Revised test in early adolescence. (4) Conclusions: Results suggest gender-specific similarities and differences in clinical and psychological features in children and adolescent males, which may require specific diagnosis and treatment.

## 1. Introduction

Eating Disorders (EDs) are severe psychiatric illnesses associated with severe medical morbidity and high rates of mortality [1,2]; EDs represent the most gendered of mental health disorders [3,4], with strong associations with femininity [3].

Historically, male presentations of EDs have been perceived as rare and unusual, resulting in the systematic underrepresentation of males in ED research [5].

Further, the notion of male ED presentation was broached in the extant literature nearly a century after the first clinical description of anorexia nervosa (AN) [1,6,7]. If in DSM-IV, diagnostic criteria were mostly tailored for the female gender [8] with the amenorrhea criterion [1,5,8]; in DSM-5 [9,10] the amenorrhea criterion was removed, and the evaluation of significantly low body weight has become more flexible and left to the appraisal of the clinician [8]. Consequently, new DSM-5 criteria allowed more eating disorders in males to be identified by a specific diagnosis instead of a residual category such as “eating disorder not otherwise specified” (EDNOS) [8,11], and the number of men diagnosed with an eating disorder increased. A significant improvement in the diagnostic definition in males is represented by the introduction in DSM-5 of the category of avoidant/restrictive food intake disorder (ARFID), a feeding and eating disorder characterized by avoidant or restrictive eating resulting in significant weight loss or failure to achieve expected weight gain, by nutritional deficiency, dependence on enteral feeding or oral nutritional supplements, and interference with psychosocial functioning. DSM-5 describes specifically three core ARFID presentations: avoidance based on the sensory characteristics of food, fear of aversive consequences associated with food intake, and lack of interest in food or eating [12]; typically, subjects affected by ARFID are more frequently males and younger than other EDs [13].

Nevertheless, research of male disordered eating behaviors is still scarce [3,14], and only in recent years literature has clarified the epidemiology of these phenomena [3]. Emerging evidence now indicates that rates of EDs in males are increasing faster than for females, and with no differences in clinical severity of symptoms [1,15,16]. In particular, males are estimated to make up 5–10% of cases of anorexia and bulimia nervosa [17] and they are generally younger than females [17,18] with a lifetime prevalence of anorexia nervosa of 0.16–0.3% [19,20,21,22], of bulimia nervosa of 0.1–0.5% [20,21,22], and of BED of 1.1–3.1% among men [20,21,22,23]. The ratio of lifetime prevalence of anorexia nervosa and bulimia nervosa in males vs. females is often reported to be equal or less than 1:10 [24], but these data probably reflect clinical under-detection among males [5] and recent epidemiological studies report higher variation for anorexia nervosa (1:3–1:12) and for bulimia nervosa (1:3–1:18) [19,20,22], while in BED the male to female rate ratio is more equal (1:2–1:6) [20,23]. The prevalence of any EDs in males was recently reported to be 1.2% at 14 years, 2.6% at 17 years, and 2.9% at 20 years [25], and in a community cohort study of adolescents the lifetime prevalence of any ED was 1.2% among males compared to 5.7% among females [26], with BED the most common diagnosis [26].

Concerning clinical features, research indicates that there are no gender differences in the age of presentation of EDs [22], including for early-onset cases [18]. Specifically, preadolescent presentation of ED in males comprises more than 1 in 4 cases in specialty clinics in Australia [18], and as many as one-third of cases in the UK [27]. Despite decades of research focusing exclusively on female populations, body image disturbances and EDs are increasingly recognized in male populations [3]. Males may have higher drive for muscularity [28], which can, in extreme cases, lead to muscle dysmorphia, a subtype of the obsessive mental disorder body dysmorphic disorder (BDD) [29]; its symptoms, such as body image disturbance, disordered eating, comorbid personality traits, and anxiety/affective disorders exhibit overlap with symptoms of eating disorders. Muscle dysmorphia’s classification has been widely debated, and alternative DSM classifications in ED have been proposed [30,31]. Nevertheless, there are still limited data on clinical presentation and risk factors for eating disorders among adolescent and young adult males [17].

In terms of clinical characteristics, cross-sectional data suggest that males with EDs are more likely to report a greater array of psychiatric comorbidities (e.g., substance use, psychotic symptoms) [5], a history of being overweight or previous obesity, and experience of being subjected to weight-related teasing [5]. Further, adolescent and young adult males with ED, especially with binge ED, generally report less shape and weight concern, drive to thinness, and body dissatisfaction than females [32,33,34,35]. Whereas body dissatisfaction in females is usually associated with a desire to be thinner, in adolescent and young adult males is centered around being “bigger” and more muscular [36,37]. An exception to this is males with anorexia nervosa who may be more likely to have concerns about thinness and not muscularity [38]. Other gendered differences include a minor engagement in males in dieting, laxative use, and self-induced vomiting than females [39,40,41], a minor reporting of eating in response to negative emotion, of experiencing a sense of loss of control when binge eating, and of restricting their food intake in response to body dissatisfaction [40,42,43]. Homosexuality has also been identified as a male-specific risk factor for the development of EDs [44,45,46] and people who identify as trans, gender non-binary or gender diverse are at two to four times greater risk of eating disorder symptoms or disordered eating behaviours than their cisgender counterparts [47].

Controversial results are reported concerning differences in overexercising [48,49]: being an athletes may be a risk factor for adolescent boys compared to females. This may be exacerbated by athletic norms associated with sports that emphasize muscularity and strength or weight control and loss [3].

Ultimately, and not less relevant, adolescent and young adult males may be less likely to seek treatment than females, also probably due to a double stigma: the stigma of suffering of a psychiatric disorder, and an additional stigma (shame, discrimination etc.) of suffering from what is commonly perceived to be a female-specific disorder [40,50,51,52]; this could contribute to misrepresenting epidemiological data and the scarcity of research on this topic.

The present research is part of a wider discussion on eating disorders in males and on the distinctive characteristics compared to females, with the aim of providing a contribution that could improve the understanding of the clinical and psychological characteristics through the use of a large cohort of preadolescents and adolescents with EDs.

Our study highlighted that even though adolescent males with EDs show similar clinical conditions to females, they are more compromised in term of general psychological suffering especially in early adolescence. Such results suggest gender-specific similarities and differences that may require specific diagnosis and treatment.

## 2. Materials and Methods

This is a single-center, observational and cross-sectional study conducted on a sample of 287 adolescents aged 6–18 years hospitalized at Child and Adolescent mental health Department- ASST Monza, University of Milano-Bicocca (Monza, Italy) from January 2014 to September 2021 for EDs (diagnosed according to DSM-5 criteria).

Exclusion criteria were intellectual disabilities (assessed through academic achievements) and neurological disorders.

Socio-demographic, clinical data and information related to the course of ED, family history and psychiatric and medical comorbidities were collected by medical records. 

Psychological profiles were analyzed for subjects older than 12 years (according to scales’ indications) through self-administered tests:

- EDI-3 (Eating Disorders Inventory, Italian version [53]): a self-report instrument to measure psychological traits typical in individuals with EDs. This test is constituted by 91 items organized into 3 eating disorder-specific scales and 9 general psychological scales relevant to, but not specific to, eating disorders. Furthermore, it includes six composite scales, one that is specific for eating-disorders (Eating Disorder Risk -EDRC-) and five that explore general integrative psychological constructs (Ineffectiveness -IC-, Interpersonal Problems -IPC-, Affective Problems -AP-, Overcontrol -OC-, and Global Psychological Maladjustment -GPCM-). The reliability coefficients of the scales range from 0.80 to 0.90, and test–retest reliability coefficients for the various composite scales are between 0.93 and 0.98.
- TAS-20 (Toronto Alexithymia Scale, Italian version): a self-report 20-item questionnaire to evaluate the alexithymia (the lack of emotional awareness) in the total level and three factors: difficulties in identifying feelings (DIF), difficulties in describing feelings (DDF), and lack of focus on internal emotional experiences (EOT). The cut-off score for determining the presence of alexithymia tract is >61 at the scale. The Italian version showed good internal consistency (Cronbach’s α of 0.75 and 0.82 in normal and clinical groups, respectively) and high test–retest reliability over 2 weeks (r = 0.86). A confirmatory factor analysis revealed the same factor structure as the original English version and adequate internal consistency of the subscales, with α coefficients equal or greater than 0.70 [54].
- SCL-90R (Symptom Checklist 90- Revised, Italian version): a self-report questionnaire to assess psychological problems and psychopathological symptoms on individuals aged 13 years and older. The original questionnaire and the one validated in Italian consists of 90 items rated on a five-point Likert scale that assess nine symptom dimensions and also provides 3 indexes: General Symptomatic Index (GSI), which discriminates subjects in a psychopathological condition and at high risk of psychiatric disorder; Positive Symptom Total (PST), which relates to the number of symptoms checked; and Positive Symptom Distress Index (PSDI), a ratio between the sum of all items and the PST. Scores between 55 and 65 are considered borderline, higher than 65 pathological. The Italian version showed good internal coherence for all subscales (α values between 0.70 and 0.96) [55].
- CDI (Children’s Depression Inventory, Italian version): a scale to assess depressive symptoms in children and adolescents aged 8–17 years. The test contains 27 items, scored from 0 to 2, and for each item, the individual had to select the statement that best describes his or her feelings over the past two weeks. The total score is the sum of the responses to all the items. The CDI uses cut-off scoring: equal to or less than 16 = no depression trait; equal to or greater than 19 = presence of a depression trait. Score of 17 or 18 = border trait. The Italian version showed good internal consistency (Cronbach’s α = 0.80) [56,57].

The-CGAS (Children’s Global Assessment Scale, Italian version) has been compiled by the clinicians for all subjects; it’s a widely used rating scale designed to measure how a child/adolescent functions psychosocially in daily life [58]. It was previously used in samples of adolescents with psychiatric morbidities [59]. The scale is subdivided into 10-point sections, each of which reports a description of the level of global functioning. The final score ranges from 1 (the most impaired level of global functioning) to 100 (the superior level of global functioning). The authors report an inter-rater reliability of 0.84, and a test–retest reliability of 0.85.

Accounting for the lack of normality, the continuous variables were expressed through median and skewness coefficients of the corresponding distribution; the categorical variables were expressed as absolute or percentage frequencies. Comparison of psychological features in the two groups were conducted through univariate analyses using the non-parametric Mann–Whitney U test. When ties were present, the analyses were performed using a modified variance. The effect sizes of the U test are reported and computed with the Wendt formula that ranges from 0 to 1. Chi-squared or Fisher’s exact tests were performed to compare categorical variables. The level of significance was set at *p* < 0.05.

Statistical analysis was performed using the SPSS 26.0 package (IBM Corp. Released 2019. IBM SPSS Statistics for Windows, Version 26.0. Armonk, NY: IBM Corp) and R version 4.1.3 (R Foundation for Statistical Computing, Vienna, Austria).

## 3. Results

In our study we included 287 adolescents with eating disorders hospitalized in our center (2014–2021), among which 27 males with EDs and 260 females with EDs.

The two groups differed for age, with males younger than females (median age in males 12.65, skew −0.271, in females 15.145, skew −1.056 U 2220, *p* = 0.0017). To take this difference and its relevance in developmental age into account, the sample has been subdivided into three subgroups: middle childhood (age 6–11), early adolescence (age 12–14) and middle adolescence (15–17), and analyses were conducted separately in each group.

No difference between males and females emerges in the three subgroups for age, BMI, last BMI before illness, deltaBMI and disease duration (Table 1, Table 2 and Table 3) except for BMI in middle childhood, where males showed a median BMI higher than females (*p* = 0.024).

The distribution of diagnosis differed in middle childhood where the 100% of males presented a diagnosis of ARFID, while the 55.6% of females of females of AR-R (*p* = 0.002) and in middle adolescents where the 66.67% of males showed a diagnosis of AN-R, and the 33.33% a diagnosis of EDNOS, while in females distribution result more heterogeneous (*p* = 0.008). Moreover, SES distribution differed in middle childhood (*p* = 0.041) with the 60% of the sample with a medium level. No other differences emerged in the other socio-demographic and medical characteristics (Table 4).

Concerning psychological scales, no differences emerged in CGAS scores in middle childhood (Table 5). In early adolescents, males showed higher scores than females in two of the three composite scale of SCL90-R: Global Severity Index (*p* = 0.006) and Positive Symptoms Total (*p* = 0.015) (Table 6). In middle adolescents no differences emerge between males and females (Table 7) in psychological scales.

## 4. Discussion

EDs characteristics and psychological profiles of children and adolescent males are still unclear and under-investigated and to our knowledge no previous studies have investigated at the same time ED features and clinical presentation and psychological profiles in a so large a cohort of subjects.

In our sample males were younger than females (median age 12.65 vs. 15.145), in line with data of literature on children and adolescents [17,18], and therefore the sample has been subdivided in three subgroups (middle childhood, early adolescents and middle adolescents) with clinical, medical and psychological features analyzed separately for each group.

Concerning the clinical presentation, which is another aspect still little investigated [17], males in middle childhood showed higher BMI, although in a condition of severe underweight, than the female counterparts, while in the other groups BMI are similar. No differences emerged in the comparison in BMI before illness, and also in deltaBMI (entity and rapidity of weight loss). These data are in contrast with previous research conducted both on children with ARFID, the diagnosis presented by the 100% of males in middle childhood in our sample (see below), where physical consequences are described more severe than in children with AN, and on adolescents and young adults that generally report a premorbid history of overweight or obesity [5,60,61].

The distribution of diagnosis in the three group is not homogeneous: in fact, in middle childhood the totality of male subjects presented a diagnosis of ARFID and in middle adolescents when 70% of males received a diagnosis of AN-R and the 30% of EDNOS, (there is just one Male diagnosed with ARFID in Early adolescence). In contrast, the diagnosis of female subjects is more heterogeneous. These data confirm previous results on the prevalence of ARFID in male children [13], but contrasts with epidemiological data on middle adolescence where BED is reported as the most common diagnosis [26]. We hypothesize that these different data could be explained by the fact that typically adolescents with BED turn first to pediatric outpatient clinics and not to ED services. They probably come to ED services in a second time, when they are older and no longer treated by Children and Adolescents Mental Services. Indeed, in our population males are only the 4.4% of Middle Adolescents, and Middle Adolescents are only 22.2% of males with ED.

Our data show that there are no statistically significant differences between males and females across the three groups for what concerns medical and psychiatric issues. Again, it is age that emerges as the most important parameter. In particular, we see a statistically significant increase (+10%) in psychiatric comorbidity in males from Middle Childhood (around 22%) to Early Adolescence (33%), and then again to Middle Adolescence (50%). In females instead, psychiatric comorbidity sees a more homogeneous distribution, with highest psychiatric comorbidity in Middle Adolescence (42%), followed by Middle Childhood (38%) and Early Adolescence (33%).

These data of increased psychiatric comorbidities in ED during adolescence are congruent with previous research but differ from epidemiological data that report a higher prevalence of comorbid substance abuse and psychotic symptoms in adolescent males [5], especially associated with a diagnosis of BED [32]. The absence of subjects with a BED diagnosis in our population is likely the reason for the discrepancy of our results from previous ones on this topic.

Furthermore, there are no statistically significant differences in overexercising across males and females.

However, our data show an increase in overexercising both in males and females from childhood to adolescence; in adolescence, males exceed 60% and females do not reach 50%, while in childhood they do not exceed 10% and 11%, respectively. The hypothesis is that the increasing use of overexercising from childhood to adolescence might be an attempt to enforce control over one’s own body in front of the pubertal changes, or/and a symptom of psychiatric comorbidities such as anxiety.

Finally, we investigated the psychological profile in early and middle adolescent males in comparison to females through self-completed scales. A first interesting aspect is that no differences between males and females, both in early and in middle adolescents, emerge in all the subscales and composite scales of EDI-3 test, suggesting that the psychological profile relating to the eating disorder is similar. This result is in contrast with Geist et al.’s study in which males endorsed lower drive for thinness and body dissatisfaction than did females [62], and with Gorrell et al. [63] who reported less severe overall ED psychopathology in males compared with females. BD and DT in EDI-3 reflect a body image disturbance (BID) more related to a third person in an allocentric perspective (the body experienced as an object). These aspects do not show significant differences between males and females; we can perhaps hypothesize that the body seen as an object is a common trait in adolescence, also favored by the overuse of social media by all adolescents, regardless of gender. It might be interesting to evaluate a BID more related to an egocentric perspective that involves interoceptive percepts about the nature and state of the body [64], especially with regard to subjects diagnosed with ARFID.

A second interesting result is that early adolescent males (but not middle adolescent males) obtained significantly higher scores in SCL-90 global indexes (GSI, PST). Higher and pathological scores in this test are considered expressions of serious and major levels of general psychological distress. This result, never described in earlier research, seems to indicate a peculiar psychopathological profile in early adolescents males than females, characterized by similar psychological profiles related to the ED but a more severe general psychopathological profile. Otherwise in middle adolescents the psychological profile does not seem to be influenced by gender, in contrast with scarce data of literature [65]. However, it is important to remember that in our population males in Middle adolescence are only six, the 4,4% of Middle adolescents with an ED.

## 5. Conclusions

In our study the differences between males and females with EDs appear to be few, and mostly correlated with age: the average age of males is indeed lower than females. This also may explain the significant prevalence of males in ARFID diagnosis, which has a well-known lower onset age and where we can see a higher proportion of male subjects compared with other EDs. Presumably, in subjects with ARFID the obvious suffering of the child expressed directly at a somatic level, and the carers’ concern, facilitate their turning to our services, sometimes after a first paediatric evaluation. In our research adolescent males—whose numbers decrease as the population’s age increases—are divided between the prevailing diagnosis of R-AN and EDNOS, while females have a more heterogeneous diagnosis distribution. Our population presents a lack of subjects with BED, an ED in which typically the male–female distribution is more balanced. On the one hand, this limits our observation powers; on the other hand, age of onset of BED is slightly older than AN or BN, typically occurring during late adolescence or young adulthood (e.g., median onset = 23.3 years) [66]. Moreover, BED is commonly perceived a less severe disorders, less impairing, with a lower awareness of disease. It is therefore less likely that subjects with BED may be referred to our services, which cater for children and adolescents until 18 years of age. It is also possible that the lack of male representation in older age depends on the phenomenon’s underestimation, connected to multiple factors. These factors include the still very pervasive belief that EDs be an exclusive female diagnosis, connected with the shame on the subjects’ part in admitting a “female” disorder, and the possibility that in male adolescents EDs exhibit a different set of symptoms, which may look similar to muscle dysmorphia.

From this perspective, it is important to continue to raise awareness of these topics, and to be particularly mindful of populations at risk (e.g., athletes). Indeed, in the last few years we saw an increase in males’ access to our services, although their numbers remain limited.

Further research should be conducted on larger samples, studying specific eating disorder with gender-specific rating scales.

## Figures and Tables

**Table 1:** Clinical features of Middle childhood Participants at admission.

	Male (N = 10)		FeMale (N = 27)				
	*Me*	*Skew*	*Me*	*Skew*	*U*	*p-Value*	*r*
BMI ^a^	14.75	1.273	13.55	0.693	69	* 0.024	0.49
Last BMI ^a^ before illness	15.85	0.958	14.97	1.019	81	0.064	0.40
ΔBMI ^a^	1.17	−1.010	1.54	0.234	111	0.412	0.18
Disease duration (months)	2.5	0.959	6	3.122	100.5	0.234	0.26

Note. ^a^ BMI = Body Mass Index. * *p* < 0.05.

**Table 2:** Clinical features of Early adolescence Participants at admission.

	Male (N = 11)		FeMale (N = 95)				
	*Me*	*Skew*	*Me*	*Skew*	*U*	*p-Value*	*r*
BMI ^a^	14.35	1.361	15.36	0.364	460	0.517	0.12
Last BMI ^a^ before illness	17.7	0.450	19.05	1.286	512	0.913	0.02
ΔBMI ^a^	4.66	−1.202	3.66	−2.352	493.5	0.764	0.06
Disease duration (months)	7	2.570	6	9.432	495.5	0.779	0.05

Note. ^a^ BMI = Body Mass Index.

**Table 3:** Clinical features of Middle adolescence Participants at admission.

	Male (N = 6)		FeMale (N = 138)				
	*Me*	*Skew*	*Me*	*Skew*	*U*	*p-Value*	*r*
BMI ^a^	16.94	−0.208	16.14	0.596	348	0.509	0.16
Last BMI ^a^ before illness	23.47	−0.224	20.74	0.977	303	0.267	0.27
ΔBMI ^a^	6.23	−0.675	4.39	1.427	329.5	0.398	0.20
Disease duration (months)	14	1.208	7	2.430	278.5	0.174	0.33

Note. ^a^ BMI = Body Mass Index.

**Table 4:** Socio-demographic and medical characteristics of Participants.

	Middle Childhood				Early Adolescence				Middle Adolescence			
	Male		Female		Male		Female		Male		Female	
	*n*	*%*	*N*	*%*	*n*	%	*N*	%	*n*	%	*N*	%
Diagnosis	Fisher’s—*p*		** 0.002		Fisher’s—*p*		0.451		Fisher’s—*p*		** 0.008	
R-AN ^a^	0	0	15	55.56	9	81.82	69	73.4	4	66.67	100	72.46
BP-AN ^b^	0	0	4	14.81	0	0	14	14.89	0	0	28	20.3
EDNOS ^c^	0	0	*1*	3.70	1	9.09	8	8.51	2	33.33	5	3.62
ARFID ^d^	8	100	7	25.93	1	9.09	3	3.2	0	0	5	3.62
Psychiatric family history	Fisher’s—*p*		0.872		Fisher’s—*p*		0.232		Fisher’s—*p*		0.574	
No	8	80	24	88.89	6	54.55	71	76.34	3	50	96	69.57
Yes	2	20	3	11.11	5	45.45	22	23.66	3	50	42	30.43
ED ^e^ family history	Fisher’s—*p*		1		Fisher’s—*p*		1		Fisher’s—*p*		1	
No	9	90	25	92.59	10	90.91	87	93.55	6	100	131	95.62
Yes	1	10	2	7.41	1	9.09	6	6.45	0	0	6	4.38
Medical comorbidities	Fisher’s—*p*		0.438		Fisher’s—*p*		0.438		Fisher’s—*p*		0.170	
No	10	100	24	88.91	10	90.91	88	95.65	4	80	130	94.2
Endocrine conditions	0	0	0	0	0	0	1	1.09	1	20	2	1.45
Malabsorption conditions	0	0	1	3.70	1	9.09	2	2.17	0	0	4	2.9
Rheumatological conditions	0	0	1	3.70	0	0	0	0	0	0	2	1.45
Metabolic disorders	0	0	0	0	0	0	1	1.09	0	0	0	0
Malabsorption and endocrine Conditions	0	0	0	0	0	0	0	0	0	0	0	0
Neurological disorders	0	0	1	3.70	0	0	0	0	0	0	0	0
Psychiatric comorbidities	Fisher’s—*p*		0.374		Fisher’s—*p*		0.128		Fisher’s—*p*		0.289	
No	7	77.78	16	61.53	6	66.67	62	68.13	3	50	80	57.96
OCD ^f^	2	22.22	1	3.85	2	22.22	2	2.20	0	0	8	5.80
Mood Disorder	0	0	1	3.85	0	0	4	4.40	0	0	12	8.70
Psychosis	0	0	1	3.85	0	0	6	6.59	2	33.34	11	7.97
Personality Disorder	0	0	0	0	0	0	6	6.59	0	0	12	8.70
Learning Disorder	0	0	1	3.85	1	11.11	1	1.10	0	0	2	1.45
Anxiety Disorder	0	0	6	23.07	0	0	6	6.59	1	16.66	5	3.62
Other disorders	0	0	0	0	0	0	4	4.40	0	0	8	5.80
Compulsive exercise	Fisher’s—*p*		1		Fisher’s—*p*		0.120		Fisher’s—*p*		0.681	
No	9	90	24	88.89	4	36.36	58	61.7	2	33.33	70	50.72
Yes	1	10	3	11.11	7	63.64	36	38.3	4	66.67	68	49.28
NSSI ^g^	Fisher’s test *p*		1		Fisher’s test *p*		0.166		Fisher’s test *p*		0.597	
No	10	100	27	100	8	72.73	82	88.17	6	100	110	79.71
Yes	0	0	0	0	3	27.27	11	11.83	0	00	28	20.29
Elimination conducts	Fisher’s—*p*		1		Fisher’s—*p*		0.195		Fisher’s—*p*		1	
No	10	100	26	96.3	8	72.73	81	87.1	5	83.33	107	77.54
Yes	0	0	1	3.7	3	27.27	12	12.9	1	16.67	31	22.46
Pharmacological Therapy	Fisher’s—*p*		0.278		Fisher’s—*p*		0.150		Fisher’s—*p*		1	
No	9	90	26	100	8	72.73	83	88.31	6	100	111	80.44
Antipsychotic	0	0	0	0	0	0	4	4.26	0	0	3	2.17
Antidepressant	0	0	0	0	1	9.09	2	2.13	0	0	9	6.52
Anxiolitic	1	10	0	0	1	9.09	1	1.06	0	0	6	4.35
Antipsychotic + antidepressant	0	0	0	0	0	0	1	1.06	0	0	2	1.45
Antipsychotic + anxiolitic	0	0	0	0	0	0	1	1.06	0	0	0	0
Antidepressant + anxiolitic	0	0	0	0	0	0	1	1.06	0	0	2	1.45
Antipychotic + antidepressant + Anxiolitic	0	0	0	0	1	9.09	1	1.06	0	0	5	3.62
NGT ^h^	Fisher’s—*p*		0.856		Fisher’s—*p*		1		Fisher’s—*p*		1	
No	9	90	23	88.46	10	90.91	86	91.49	6	100	132	95.65
Yes	1	10	3	11.54	1	9.09	8	8.51	0	0	6	4.35
Socio-Economic Status	Fisher’s—*p*		* 0.041		Fisher’s—*p*		0.371		Fisher’s—*p*		0.797	
Low	1	10	3	12.5	4	36.37	16	17.78	2	33.33	24	18.46
Medium–Low	3	30	5	20.83	1	9.09	20	22.22	2	33.33	27	20.77
Medium	6	60	4	16.67	3	27.27	23	25.56	1	16.665	31	23.85
Medium–High	0	0	8	33.33	1	9.09	22	24.44	1	16.665	39	30
High	0	0	4	16.67	2	18.18	9	10	0	0	9	6.92

Note. ^a^ R-AN: restrictive anorexia nervosa; ^b^ BP-AN: binging-purging type anorexia nervosa; ^c^ EDNOS: Eating Disorder Not Otherwise Specified; ^d^ ARFID: Avoidant Restrictive Food Intake Disorder; ^e^ OCD: Obsessive Compulsive Disorder; ^f^ ED: Eating Disorder; ^g^ NSSI: Non-Suicidal Self-Injury; ^h^ NGT: Nasogastric Tube. * *p* < 0.05. ** *p* < 0.01.

**Table 5:** Differences in CGAS scores in Middle childhood.

	Male		Female				
	*Me*	*Skew*	*Me*	*Skew*	*U*	*p-Value*	*r*
C-GAS ^1^	60	−0.458	60	0.342	89	0.891	0.03

Note. ^1^ C-GAS = Children’s Global Assessment Scale.

**Table 6:** Differences in psychological features in Early adolescents.

	Male		Female				
	*Me*	*Skew*	*Me*	*Skew*	*U*	*p-Value*	*r*
EDI-3 ^1^							
DT ^2^	85	−0.686	75	−0.490	235	0.810	0.05
B ^3^	73	−1.087	56.5	−0.006	112	0.508	0.16
BD ^4^	60.5	−0.105	69	−0.583	211.5	0.687	0.09
LSE ^5^	72.5	−1.035	67	−0.335	239.5	0.875	0.03
PA ^6^	68	−0.755	71	−0.380	234	0.969	0.01
II ^7^	72.5	−0.762	81	−0.731	223	0.644	0.10
IA ^8^	36	0.436	66	−0.468	174	0.155	0.31
ID ^9^	79.5	−0.884	76	−0.986	218.5	0.735	0.07
ED ^10^	62	−0.500	69	−0.463	200	0.679	0.09
P ^11^	66.5	−0.454	70	−0.454	256	1	0
A ^12^	66.5	−0.770	64	−0.316	208	0.744	0.07
MF ^13^	80	−0.981	59	−0.176	189	0.330	0.21
EDRC ^14^	74	−0.606	68	−0.621	202	0.603	0.11
IC ^15^	74	−0.808	71	−0.516	232	0.822	0.05
IPC ^16^	58.5	−0.200	74	−0.683	204.5	0.542	0.13
APC ^17^	71	−0.783	76	−0.901	243	0.985	<0.01
OC ^18^	71	−0.962	61	−0.479	204.5	0.638	0.10
GPMC ^19^	79.5	−0.289	75	−0.997	147.5	0.374	0.20
TAS-20 ^20^	66	−0.390	59	−0.442	278.5	0.532	0.13
SCL-90R ^21^							
GSI ^22^	69	−0.777	49.25	0.325	125	** 0.006	0.57
PST ^23^	65	−0.054	54.5	−0.290	143.5	* 0.015	0.50
PSDI ^24^	52	0.606	51.25	0.345	246.5	0.486	0.14
CDI ^1^	17	0.823	17	0.305	307	0.902	0.03
C-GAS ^2^	54.5	0.875	55	0.302	325.5	0.498	0.13

Note. ^1^ Eating Disorder Inventory; ^2^ DT = Drive to Thinness; ^3^ B = Bulimia; ^4^ BD = Body Dissatisfaction; ^5^ LSE = Low Self-Esteem; ^6^ PA = Personal Alienation; ^7^ II = Interpersonal Insecurity; ^8^ AI = Interpersonal Alienation; ^9^ ID = Interoceptive Deficits; ^10^ ED = Emotional Disregulation; ^11^ P = Perfectionism; ^12^ A = Ascetism; ^13^ MF = Maturity Fear; ^14^ EDRC = Eating Disorder Risk Composite; ^15^ IC = Ineffectiveness Composite; ^16^ IPC = Interpersonal Problems Composite; ^17^ APC = Affective Problems Composite; ^18^ OC = Overcontrol Composite; ^19^ GPMC = General Psychological Maladjustment Composite; ^20^ TAS-20 = Toronto Alexithymia Scale, ^21^ SCL-90R = Symptom Check List-90 Revised, ^22^ GSI = Symptoms Check List-90 Revised -Global Severity Index, ^23^ PST = Symptoms Check List-90 Revised -Positive Symptoms Total, ^24^ PSDI = Symptoms Check List-90 Revised -Positive Symptom Distress Index. ^1^ CDI = Children’s Depression Inventory; ^2^ C-GAS = Children’s Global Assessment Scale.* *p* < 0.05. ** *p* < 0.01.

**Table 7:** Differences in psychological tests in Middle adolescents.

	Male		Female				
	*Me*	*Skew*	*Me*	*Skew*	*U*	*p-Value*	*r*
*EDI-3* ^1^							
DT ^2^	73	−0.519	83	−0.863	200	0.638	0.14
B ^3^	73	−0.680	61	−0.064	112.5	0.962	0.02
BD ^4^	44	0.537	77	−0.628	170	0.322	0.29
LSE ^5^	84	−0.619	79	−0.867	169	0.894	0.05
PA ^6^	58.67	−0.829	75	−0.796	224	0.314	0.27
II ^7^	69.5	−0.086	77	−0.869	211	0.719	0.11
IA ^8^	32.5	0.647	69	−0.422	148	0.205	0.37
ID ^9^	62.33	−0.912	82	−0.884	188	0.146	0.38
ED ^10^	64.67	−0.744	66.83	−0.476	299	0.941	0.02
P ^11^	81.5	−0.615	70	−0.452	157.5	0.258	0.33
A ^12^	62.67	−0.847	69	−0.966	234	0.378	0.23
MF ^13^	75	−0.848	59	−0.248	202	0.642	0.14
EDRC ^14^	59	−0.201	76	−0.719	187.5	0.531	0.18
IC ^15^	60	−0.912	79	−1.049	208.5	0.231	0.32
IPC ^16^	58.5	−0.079	77	−0.964	188.5	0.480	0.21
APC ^17^	55.33	−0.682	78	−1.089	189.5	0.152	0.38
OC ^18^	72.67	0.752	72.83	−0.960	284	0.794	0.07
GPMC ^19^	62	−0.908	77	−0.968	231	0.358	0.24
*TAS-20* ^20^	60	0.331	61	−0.300	225.5	0.817	0.07
*SCL-90R* ^21^							
GSI ^22^	60	−0.195	55.5	0.060	194.5	0.715	0.11
PST ^23^	57.25	−0.297	56	−0.426	208	0.853	0.05
PSDI ^24^	53.25	0.035	53	0.182	205	0.840	0.06
*CDI* ^25^	15	−0.689	20	0.120	122	0.095	0.49
*C-GAS* ^26^	56.5	0.249	55	0.131	246	0.914	0.03

Note. ^1^ Eating Disorder Inventory; ^2^ DT = Drive to Thinness; ^3^ B = Bulimia; ^4^ BD = Body Dissatisfaction; ^5^ LSE = Low Self-Esteem; ^6^ PA = Personal Alienation; ^7^ II = Interpersonal Insecurity; ^8^ AI = Interpersonal Alienation; ^9^ ID = Interoceptive Deficits; ^10^ ED = Emotional Disregulation; ^11^ P = Perfectionism; ^12^ A = Ascetism; ^13^ MF = Maturity Fear; ^14^ EDRC = Eating Disorder Risk Composite; ^15^ IC = Ineffectiveness Composite; ^16^ IPC = Interpersonal Problems Composite; ^17^ APC = Affective Problems Composite; ^18^ OC = Overcontrol Composite; ^19^ GPMC = General Psychological Maladjustment Composite; ^20^ TAS-20 = Toronto Alexithymia Scale, ^21^ SCL-90R = Symptom Check List-90 Revised, ^22^ GSI = Symptoms Check List-90 Revised -Global Severity Index, ^23^ PST = Symptoms Check List-90 Revised -Positive Symptoms Total, ^24^ PSDI = Symptoms Check List-90 Revised -Positive Symptom Distress Index. ^25^ CDI = Children’s Depression Inventory; ^26^ C-GAS = Children’s Global Assessment Scale.

## Data Availability

Not applicable.
